# Gender differences among Swedish COPD patients: results from the ARCTIC, a real-world retrospective cohort study

**DOI:** 10.1038/s41533-019-0157-3

**Published:** 2019-12-10

**Authors:** Karin Lisspers, Kjell Larsson, Christer Janson, Björn Ställberg, Ioanna Tsiligianni, Florian S. Gutzwiller, Karen Mezzi, Bine Kjoeller Bjerregaard, Leif Jorgensen, Gunnar Johansson

**Affiliations:** 1 Department of Public Health and Caring Sciences, Family Medicine and Preventive Medicine, Uppsala University, Uppsala, Sweden; 2 Department of Pulmonary Medicine, Work Environment Toxicology, Karolinska Institutet, Stockholm, Sweden; 3 Department of Medical Sciences: Respiratory, Allergy and Sleep Research, Uppsala University, Uppsala, Sweden; 4 Department of Social Medicine, Health Planning Unit, Faculty of Medicine, University of Crete, Crete, Greece; 5 Novartis Pharma AG, Basel, Switzerland; 6 IQVIA Solutions, Copenhagen, Denmark

**Keywords:** Health care, Disease prevention

## Abstract

The present study aimed to generate real-world evidence regarding gender differences among chronic obstructive pulmonary disease (COPD) patients, especially as regards the diagnosis and outcomes in order to identify areas for improvement and management and optimize the associated healthcare resource allocation. ARCTIC is a large, real-world, retrospective cohort study conducted in Swedish COPD patients and a matched reference population from 52 primary care centers in 2000–2014. The incidence of COPD, prevalence of asthma and other comorbidities, risk of exacerbations, mortality rate, COPD drug prescriptions, and healthcare resource utilization were analyzed. In total, 17,479 patients with COPD were included in the study. During the study period, COPD was more frequent among women (53.8%) and women with COPD experienced more exacerbations vs. men (6.66 vs. 4.66). However, the overall mortality rate was higher in men compared with women (45% vs. 38%), but no difference for mortality due to COPD was seen between genders over the study period. Women seemed to have a greater susceptibility to asthma, fractures, osteoporosis, rheumatoid arthritis, rhinitis, depression, and anxiety, but appeared less likely to have diabetes, kidney diseases, and cardiovascular diseases. Furthermore, women had a greater risk of COPD-related hospitalization and were likely to receive a significantly higher number of COPD drug prescriptions compared with men. These results support the need to reduce disease burden among women with COPD and highlight the role of healthcare professionals in primary care who should consider all these parameters in order to properly diagnose and treat women with COPD.

## Introduction

Chronic obstructive pulmonary disease (COPD) is a progressive disorder characterized by airflow limitation and is a major cause of chronic morbidity and mortality across the world. Approximately 65 million people suffer from COPD and 3 million deaths occur from it annually, making it the third leading cause of death globally.^1^ The importance and significance of COPD management has been well documented.^2^

Until some years ago, COPD diagnosis has in many cases been neglected in women as it was considered a disease that primarily affects men. According to some of the earlier reports, there was either no gender disparity in undiagnosed COPD^3^ or women were at the greater risk of undiagnosed COPD.^4,5^ However, recent studies demonstrated that the prevalence of COPD is similar among men and women.^6–11^ A study by Mamary et al.^12^ reported that men were more prone to have underdiagnosis of COPD than women. Furthermore, mortality among women due to COPD is higher than from many of the most commonly recognized forms of cancer.^10^ In a Swedish study, COPD mortality among women increased during 1999–2009, and life expectancy in the COPD population was 9.4 years lower for women (vs. 7.4 years lower in men) compared with that of the average Swedish population.^13^ Several findings also suggested that health status and quality of life is more impaired in women than in men with COPD.^14–19^

The above-mentioned findings reflect the increased incidence of smoking among females over the past decades in several countries. Although the smoking prevalence in Sweden has decreased during the period of 2006–2016, since many years it is been higher for women than men (9.6% vs. 8.0% in 2016).^20^ However, in most countries, women smoke less than men, suggesting that females may be more severely affected by COPD than males and are more susceptible to the harmful effects of smoking, thereby developing COPD more easily than males.^21^ In a large population-based study, female gender was associated with reduction in lung function and more severe disease among COPD patients with early onset of disease or low tobacco exposure.^22^ According to a systematic review, the annual decline in forced expiratory volume in 1 s was faster in female smokers compared with male smokers despite they smoke less.^23^

Findings from mainly observational studies point to a gender-specific susceptibility and morbidity, and it is important to generate real-world evidence to better understand the gender aspects of COPD. In Sweden, most patients with COPD are managed in primary care. Therefore, the aim of this study was to provide real-world evidence on gender differences with respect to diagnosis and outcomes among COPD patients in the Swedish primary care setting and to identify areas for improvement and management of such patients.

## Results

### Patient demographics

Of the identified 18,586 eligible patients with a COPD diagnosis listed in electronic medical records (EMRs), 291 patients were excluded because they were diagnosed with COPD before 40 years of age. Following case–control matching, a total of 17,479 patients with COPD (International Classification of Diseases, tenth revision [ICD-10] code: J44) were included under cases and they were compared with 84,455 age- and gender-matched controls. The patients were followed for a mean (standard deviation) duration of 12.4 (4.6) years. Patient demographics of the COPD patients and the reference population stratified by gender are summarized in Table 1. In the COPD cohort, the mean age was 68.2 years for females and 68.9 years for males. The proportion of females (54.4%) was higher compared with males (45.6%). At baseline, women were younger and had a lower Charlson comorbidity index (CCI) value compared with men. Prevalence of asthma was also significantly higher in women (*p* < 0.0001). Furthermore, although women had a higher number of primary care contacts, they had fewer overnight stays than men. The healthcare resource utilization data included both COPD and non-COPD, that is, comorbidity-related events. A significantly higher number of women used inhaled corticosteroids (ICSs) and oral corticosteroids (OCSs) compared to men. However, no significant difference was observed with respect to body mass index between the genders in both COPD and reference population.

**Table 1:** Patient demographics of the COPD and reference groups stratified by gender.

Variable	Females, COPD (*N* = 9506)	Males, COPD (*N* = 7973)	*p* value	Females, ref. population (*N* = 47,744)	Males, ref. population (*N* = 36,711)	*p* Value
Age (years), mean (SD)^a^	68.19 (11.4)	68.87 (10.7)	<0.0001	65.2 (12.5)	64.7 (11.1)	<0.0001
Body mass index (kg/m^2^), mean (SD)	25.92 (5.9)	26.24 (5.0)	0.0744	27.47 (5.6)	27.23 (4.2)	0.1807
CCI value^b^, mean	1.62	1.78	<0.0001	1.30	1.37	<0.0001
Healthcare utilization^a^						
Outpatient visits per year, mean ± SD	1.74	1.82	0.17	1.54	1.44	<0.0001
Primary care visits per year, mean ± SD	10.39	9.46	0.0006	5.12	4.45	<0.0001
Overnight stays, *n* (%)	3148 (33.12)	2894 (36.30)	<0.0001	10,869 (22.77)	8628 (23.50)	0.01
Patients with outpatient hospital visits, *n* (%)	5740 (60.38)	4835 (60.64)	0.73	28,290 (59.25)	20,367 (55.48)	<0.0001
Comorbidities^a,c^, *n* (%)						
Respiratory diseases (including COPD and asthma)	3613 (38.01)	2769 (34.73)	<0.0001	6774 (14.19)	4376 (11.92)	<0.0001
Cardiovascular diseases	3417 (35.95)	3319 (41.63)	<0.0001	10,257 (21.48)	9315 (25.37)	<0.0001
Hypertensive diseases	2170 (22.83)	1849 (23.19)	0.57	6418 (13.44)	5275 (14.37)	0.0001
Asthma	1554 (16.35)	977 (12.25)	<0.0001	2649 (5.55)	1428 (3.89)	<0.0001
Any cancer	1180 (12.41)	1121 (14.06)	0.0013	5179 (10.85)	3875 (10.56)	0.1739
Other forms of heart diseases	1138 (11.97)	1366 (17.13)	<0.0001	2539 (5.32)	2849 (7.76)	<0.0001
Fractures	678 (7.13)	448 (5.62)	<0.0001	2734 (5.73)	1335 (3.64)	<0.0001
Depression	649 (6.83)	313 (3.93)	<0.0001	1510 (3.16)	668 (1.82)	<0.0001
Diabetes, type II	546 (5.74)	707 (8.87)	<0.0001	1745 (3.65)	2114 (5.76)	<0.0001
Anxiety	455 (4.79)	243 (3.05)	<0.0001	956 (2.00)	439 (1.20)	<0.0001
Rheumatoid arthritis	204 (2.15)	97 (1.22)	<0.0001	624 (1.31)	184 (0.50)	<0.0001
Diabetes, type I	160 (1.68)	180 (2.26)	0.006	677 (1.42)	743 (2.02)	<0.0001
Kidney disease	114 (1.20)	209 (2.62)	<0.0001	259 (0.54)	340 (0.93)	<0.0001
Lung cancer	88 (0.93)	88 (1.10)	0.2404	99 (0.21)	82 (0.22)	0.6180
Polymyalgia rheumatica	81 (0.85)	40 (0.50)	0.0054	281 (0.59)	101 (0.28)	<0.0001
Nasal polyps	39 (0.41)	53 (0.66)	0.0206	124 (0.26)	218 (0.59)	<0.0001
Medication use^c^, *n* (%)						
Inhaled corticosteroids	2623 (27.59)	1816 (22.78)	<0.0001	5550 (11.62)	2892 (7.88)	<0.0001
Oral steroids	1897 (19.96)	1219 (15.29)	<0.0001	4028 (8.44)	2222 (6.05)	<0.0001

*ATC* anatomical therapeutic chemical, *CCI* Charlson comorbidity index, *COPD* chronic obstructive pulmonary disease, *ICD* International Classification of Diseases, *Ref*. reference, *SD* standard deviation

^a^Age at index date; comorbidities and healthcare utilization and medication use 2 years before the index date

^b^CCI is a method of categorizing comorbidities of patients based on the ICD diagnosis codes found in administrative data, such as hospital abstracts data. Each comorbidity category has an associated weight (from 1 to 6) based on the adjusted risk of mortality or resource use, and the sum of all the weights results in a single comorbidity score for a patient. The higher the score, the more likely the predicted outcome will result in mortality or higher resource use

^C^ICD-10 codes and ATC codes are reported in Supplementary Tables 1 and 2, respectively

### COPD incidence

COPD was more frequent among females than males during the observation period. The percentage of incident distribution for females had been fairly constant at 53.8%. However, a small downward trend was observed among females towards the later years of the observation period.

### Presence of asthma (ICD-10: J45/J46)

Of the 17,479 COPD patients, 6026 (34.5%) patients had a diagnosis code for asthma at any time point during the study. Of these 6026 patients, 1924 (31.9%) patients had an asthma diagnosis before the index date, while 1272 patients (21.1%) had an asthma diagnosis 0 to 2 years after the index date and 417 (6.92%) patients had an asthma and COPD diagnosis on the same date.

**Fig. 1:**
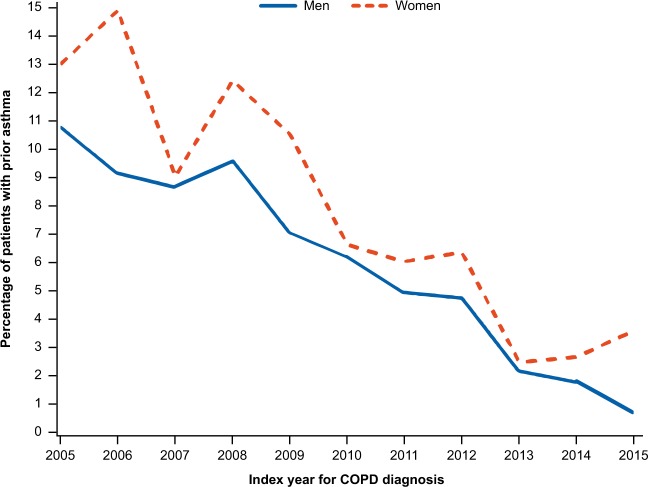
Calendar year trend in asthma before COPD diagnosis. COPD chronic obstructive pulmonary disease.

Before the index date, the asthma diagnosis showed marked difference between genders; more females than males had asthma (12.3% vs. 9.4%; *p* < 0.0001). Towards the later years of the observation period, the percentage of incident patients with the asthma had decreased significantly in both genders (Fig. 1).

Furthermore, no significant difference was observed between males and females in the time period from the asthma diagnosis to the index date (difference: 3.1 years; *p* = 0.9284).

### Comorbidities

The frequency of all comorbidities was higher in the COPD population compared with the reference population when analyzed 3 years before and 3 years after the index date. A gender difference was observed in some of the comorbidities in both COPD and reference populations, and the magnitude of this difference was generally observed to be higher in the COPD population compared with the reference population.

The comorbidities that were more prevalent in females included asthma, fractures, osteoporosis, rheumatoid arthritis, rhinitis, depression, and anxiety. The comorbidities that were more prevalent in males included type I and type II diabetes, kidney diseases, and cardiovascular diseases. The comorbidities where no gender difference was observed included respiratory diseases (excluding COPD and asthma: ICD-10 code J41–J45), polymyalgia rheumatica, nasal polyps, any cancer, lung cancer, dementia, and hyperlipidemia.

### Exacerbations and mortality

**Fig. 2:**
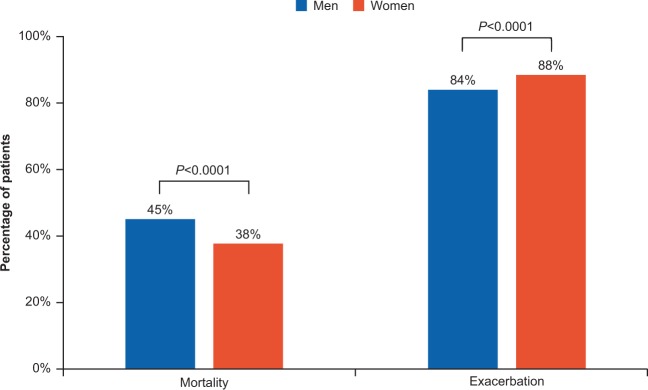
Mortality and exacerbations during the study period.

**Fig. 3:**
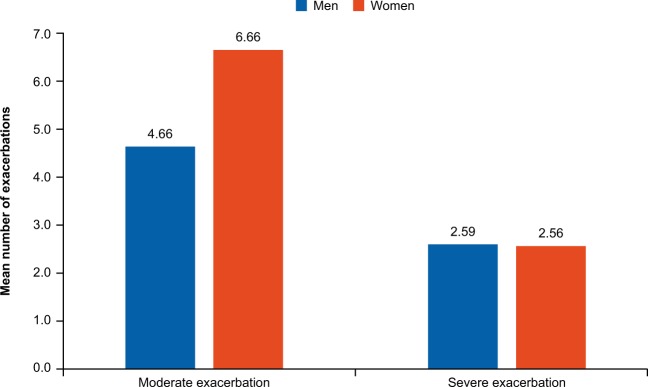
Number of moderate and severe exacerbations.

During the follow-up period, women exacerbated more frequently than men as indicated by the significantly higher frequency of exacerbations throughout the study period (Fig. 2). Time-to-first-exacerbation analysis revealed that women had a 12% higher risk of an earlier exacerbation than men (hazard ratio; HR [95% confidence interval; CI]: 1.12 [1.09–1.16], *p* < 0.0001). In terms of severity, women had more moderate exacerbations (*p* < 0.0001), while no difference in severe exacerbations (*p* = 0.60) was observed on comparison with men (Fig. 3).

However, the mortality rate was found to be significantly higher in men compared to women (Fig. 2). Time from first COPD diagnosis to death is significantly longer for women than men (HR [95% CI]: 0.78 [0.74–0.81], *p* < 0.0001).

It was observed that from COPD diagnosis to death, females live an average of 2 years longer than males. Median survival time from COPD diagnosis to death in men (9.0 years, 95% CI: 8.75–9.30) was shorter than that in women (11.1 years, 95% CI: 10.8–11.5).

**Fig. 4:**
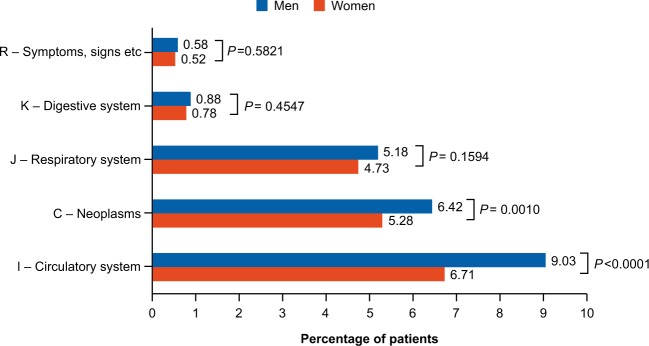
Five most common reasons of mortality among COPD patients. COPD chronic obstructive pulmonary disease.

The five most common reasons of mortality among COPD patients stratified by gender are shown in Fig. 4. During the study period, it was seen that mortality due to neoplasms and circulatory system was significantly higher among males than females. However, no difference was observed for mortality due to COPD between genders (males: *n* = 345 ± 4.01; females: *n* = 387 ± 3.87; *p* = 0.62). The detailed results are reported in Supplementary Table 3.

### Healthcare resource utilization

Males had a higher number of hospitalizations, more nights at hospital, and more outpatient visits to hospitals than females for all diseases (COPD and non-COPD, i.e., comorbidities). However, when only COPD-related incidents were analyzed, females had a higher number of hospitalizations, more nights at hospital, and more outpatient visits to hospitals (Table 2).

**Table 2:** Hospital visits per year for COPD patients during the study period.

Variable	Mean (95% CI)		*p* Value
	Male	Female	
All diagnosis			
No. of hospitalizations per year	1.43 (1.35–1.51)	1.17 (1.10–1.24)	<0.0001
No. of outpatient visits per year	2.34 (2.24–2.44)	2.16 (2.07–2.25)	0.008
No. of nights at hospital per year, all diagnosis	9.62 (9.09–10.16)	8.92 (8.42–9.42)	0.06
COPD related			
No. of hospitalizations per year	0.14 (0.12–0.16)	0.17 (0.16–0.19)	0.007
No. of outpatient visits per year	0.12 (0.11–0.14)	0.14 (0.13–0.15)	0.15
No. of nights at hospital per year	0.85 (0.70–0.99)	1.21 (1.08–1.34)	0.0002

*CI* confidence interval, *COPD* chronic obstructive pulmonary disease

There was no difference between genders for the number of visits to primary care, neither for nurse visits (mean [95% CI]: men, 8.30 [8.01–8.60]; women, 8.53 [8.26–8.81]; *p* = 0.2633) nor physician visits (mean [95% CI]: men, 6.89 [6.58–7.20]; women, 6.74 [6.45–7.03]; *p* = 0.4957). Data for primary care contacts cannot be stratified into COPD related and non-COPD related due to the structure of the primary care records.

Annual direct healthcare utilization costs in 2013 were slightly higher for men than for women (€11,054 vs. €10,173). The indirect cost (loss of income) was higher for the male patients (€13,995/year) than for the female patients (8711/€year) compared with the income in the reference population, with the difference between gender of €5284/year as a mean value for the 2006–2013 period, mostly reflecting the higher income among men.

### COPD drug prescriptions

Results suggested that females receive significantly higher number of COPD drug prescriptions (except long-acting β_2_-agonist [LABA]/long-acting muscarinic antagonist [LAMA] fixed combinations) than males (Table 3).

**Table 3:** COPD drug prescriptions^a^.

Drug	Mean no. of prescriptions per year (95% CI)		*p* Value
	Male	Female	
ICS	0.36 (0.33–0.39)	0.49 (0.46–0.52)	<0.0001
OCS	0.79 (0.73–0.86)	1.02 (0.95–1.08)	<0.0001
SABA	1.05 (0.99–1.12)	1.39 (1.33–1.46)	<0.0001
LAMA	1.42 (1.37–1.47)	1.62 (1.58–1.67)	<0.0001
LABA	0.30 (0.28–0.33)	0.40 (0.37–0.42)	<0.0001
LABA/LAMA fixed combinations	0.01 (0.01–0.01)	0.01 (0.01–0.01)	0.6511
LABA/ICS fixed combinations	1.03 (0.99–1.07)	1.16 (1.13–1.20)	<0.0001

*CI* confidence interval, *COPD* chronic obstructive pulmonary disease, *ICS* inhaled corticosteroids, *LABA* long-acting β_2_-agonist, *LAMA* long-acting muscarinic antagonist, *OCS* oral corticosteroids, *SABA* short-acting β_2_-agonist

^a^ATC codes are reported in Supplementary Table 2

## Discussion

The results of this study suggested a closing of the gap in COPD prevalence among both genders. Also, the burden of comorbidities including asthma and the risk of exacerbations was higher in women. Furthermore, COPD-related hospital visits and use of COPD drug prescriptions were higher in women than in men. In contrast, the survival rate was better among women compared to men, but no difference was observed for mortality due to COPD between genders.

In alignment with these findings, a considerable number of studies have indicated that COPD prevalence is now similar among men and women.^6–11^ This is likely due to dramatic increase in smoking rates among women in some regions of the world,^24^ including Sweden, where smoking rates are higher among women vs. men.^20^

In addition, the prevalence of asthma was significantly higher in women than in men, and these findings are concordant with previously published studies.^25,26^ One reason for this might be due to the fact that women with COPD have been more likely to be misdiagnosed with asthma than men.^5^ However, during the later years of the observation period, the proportion of incident patients with asthma had significantly decreased for both genders, which might be reflecting the improved precision in COPD diagnosis over time. Moreover, no significant difference among men and women was observed in terms of time period from diagnosis of asthma to the index date, indicating no gender difference in the diagnostic procedure. In contrast to our results, a study by Mamary et al.^12^ reported that COPD was more likely to be underdiagnosed in men compared to women. The reason stated by the author was that women report symptoms earlier and are more likely to utilize healthcare resources than men.^12^

According to a Danish study, up to 28% variation in healthcare expenditures can be caused by the presence of comorbidities, while only 5% of such variation can be caused by other demographic characteristics such as age and sex.^27^ Our findings suggest that the prevalence of comorbidities such as depression, fracture, osteoporosis, rheumatoid arthritis, rhinitis, and anxiety was higher in women than in men. On the other hand, the incidence of type I and type II diabetes, kidney diseases, and cardiovascular diseases was higher among men. These observations are in alignment with earlier published literature.^15,16,28–31^ Depression and anxiety are among the strongest predictors of impaired quality of life,^32^ and anxiety among COPD patients can lead to the vicious cycle of breathlessness and anxiety known as “dyspnea–anxiety–dyspnea cycle,” which means that patients’ emotional response to breathlessness result in intensification of their perception of breathlessness.^33^ Hence, it is very important for healthcare professionals to be knowledgeable of and take into account gender differences regarding comorbidities when managing patients with COPD.

In alignment with published literature, COPD exacerbations occur more frequently among women.^10^ This can be attributed to the greater prevalence of airway hyperresponsiveness in women, which is a significant predictor of lung function decline and also results in increased susceptibility to harmful effects of smoking among women.^34^ As the risk of exacerbations is higher in women, COPD-related healthcare resource utilization is also higher, indicating that women may benefit from closer monitoring of their exacerbation risk. Therefore, healthcare professionals should be more aware of these differences and try to be more prone to investigate, treat, and follow-up women with COPD.

On the other hand, we found that survival in women was better vs. men, which was consistent with findings from other studies.^21,35^ Findings from the PATHOS study conducted in Sweden revealed that COPD mortality in women increased from 1999 to 2009, and life expectancy among COPD patients was 9.4 years lower in women (vs. 7.4 years lower in men) compared with the average Swedish population.^13^ Among the five most common reasons of mortality, a significantly higher rate was observed for mortality due to neoplasms and circulatory system among males compared to females; however, there was no difference for mortality due to COPD between genders.

In general, women used more medications for COPD, and this trend might be due to various reasons, including more exacerbations, more respiratory symptoms and impaired quality of life, and higher prevalence of asthma compared to men. As asthma is more frequent in women, the study results demonstrated that women received significantly more prescriptions for ICS and LABA/ICS fixed-dose combinations compared to men. It was also observed that significantly higher proportion of women are prescribed OCS than men and this can be because of the higher exacerbation rate in women. Use of ICS is known to further increase the risk of fractures, particularly in women with COPD who are already at higher risk of osteoporosis compared with men.^36^ The long course use of OCS in patients with acute exacerbation of COPD is associated with increased risk of pneumonia hospitalization and all-cause mortality within 1-year period from the start of treatment.^37^ Hence, treatment with ICS and OCS in women with COPD should be carefully assessed.

The present study has several important strengths. The large sample size of the study comprised patients across the whole COPD classification spectrum. Moreover, the primary care setting and the real-world study design adequately reflects the general population and clinical practice in Sweden. Furthermore, measurement of the comprehensive range of outcomes over the extended assessment period (2000–2014) generated robust data on gender differences between COPD and non-COPD patients. Data quantification in this study can allow decision makers to identify gender-specific differences in COPD risk and outcomes and analyze how integrated screening and treatment strategies can be devised to achieve better outcomes for millions of women with COPD worldwide.

Nevertheless, this study also has certain limitations. As this was a retrospective study, the potential for bias and confounding cannot be excluded. Although all patients had physician-diagnosed COPD, the accuracy of COPD diagnosis and the severity of disease could not be verified. In addition, use of medications was based on prescription claims, while patients’ actual adherence to treatment remains unknown. Moreover, this study was conducted only in Swedish patients, and it is therefore uncertain whether these findings can be extrapolated to a more diverse group of patients and to other healthcare systems. Furthermore, the reference population was identified from primary care centers rather than from healthy individuals in the general population.

In conclusion, our findings demonstrated that COPD is now equally prevalent among men and women, thus imposing a significant healthcare disease burden among women. Despite a lower mortality rate, women had higher prevalence comorbidities including asthma and a higher risk of exacerbations, leading to more utilization of COPD drugs and COPD-related healthcare resources. In daily clinical practice, healthcare professionals in primary care play a pivotal role and should consider all these parameters in order to properly diagnose and treat women with COPD. Additional research is warranted to understand the gender-specific differences and to formulate gender-targeted strategies for prevention and treatment of COPD in both women and men. The focus should also be on recognizing and treating comorbidities together with interventions that directly target COPD.

## Methods

### Study design

ARCTIC was a large, real-world, retrospective, Swedish cohort study conducted in 18,586 eligible, primary care, COPD patients. Ethical approval for the study was obtained from the local Ethical Regional Board in Uppsala, Sweden, on 11 December 2014 (number: 2014-397), for accessing the National Health Register and for recruiting primary care centers to the study. An amendment specifying additional analysis was approved by the Ethical Regional Board in Uppsala on 6 October 2017. Data from all records were de-identified, and therefore, patient consent was not required by the ethics committee.

Following ethical approval, EMR data for COPD patients between 2000 and 2014 from 52 primary care centers across Sweden were collected using an established software system (Pygargus Customized eXtraction Program) and included age and gender, prescriptions (according to the World Health Organization Anatomic Therapeutic Chemical [ATC] codes), doctor’s diagnoses (according to the ICD-10 codes), spirometry measurements, laboratory tests, healthcare professional visits, and referrals. The centers covered urban and rural sites of varying sizes across Sweden. EMR data were linked by the Swedish National Board of Health and Welfare using individual patient identification (ID) numbers to National Registry data sources (patient IDs were pseudonymized). These data sources included (i) the Longitudinal Integration Database for Health Insurance and Labor Market Studies, which contains socio-demographic data, including educational level, marital status and family situation, occupational status, retirement and economic compensation, and social benefits; (ii) the National Patient Register, which contains data related to diagnosis from secondary care (ICD-10 code and associated position), including surgery, gender, age, region, hospital visits, specialty visits, hospital admissions, discharges, medical procedures, and surgeries performed in inpatient and outpatient specialist settings; (iii) the National Prescription Register (from 2005), which contains the full details of all dispensed medications (ATC codes) from both primary and secondary care, including brand name, prescription date, dose, strength, pack size, specialty of the prescriber, and costs associated with the drug prescription; and (iv) the Cause of Death Register, which holds information related to sex, date of death, and the underlying cause of death.

### Study patients

The study population consisted of patients aged ≥40 years who had received either a physician’s diagnosis of COPD (ICD-10 code: J44) in primary care (EMR database) or a physician’s diagnosis of asthma (ICD-10 code: J45/J46) in primary care that was later verified as COPD, or COPD was added to the asthma diagnosis in the hospital setting according to the National Patient Register. An age- and gender-matched reference population was selected from the primary care centers, excluding those who had a diagnosis of COPD and/or asthma. The age and gender were matched for overall population and not at the subgroup level. Therefore, differences in age could be observed when analyzed by gender subgroups.

### Outcomes

Difference between genders was evaluated with respect to the incidence of COPD; prevalence of asthma and comorbidities; risk of exacerbations and severity of exacerbations; mortality rate, duration from the index date to death and reason for mortality, healthcare resource use and costs; and the number of COPD drug prescriptions. An overall measure of comorbidities was calculated by the CCI. CCI is a method of categorizing comorbidities of patients based on the ICD diagnosis codes found in primary care medical records and register data from secondary care. Each comorbidity category has an associated weight (from 1 to 6), based on the adjusted risk of mortality or resource use, and the sum of all the weights results in a single comorbidity index for a patient. The higher the index, the more likely the predicted outcome will result in mortality or higher resource use.^38^ Prevalence of the most common comorbidities at 3 years before and after the first COPD diagnosis was compared between genders for both COPD patients and non-COPD patients (reference population).

“Moderate exacerbations” were defined as treatment with systemic corticosteroids (H02AB) or antibiotics (J01AA, J01CA) or both (but no hospitalization). “Severe exacerbations” were defined as COPD-related hospitalizations (J44 in primary position or J44.0/J44.1 in secondary) or emergency visits (J44.0/J44.1 in outpatient hospital care). Recurrent exacerbations occurring within 14 days were considered as one unique event.

“Hospital nights” was defined as the sum of overnight stays at a hospital per patient. “Hospitalizations” was the number of times that a patient has been admitted to a hospital regardless of the length of stay. “Outpatient visit” was defined as the visit to a hospital that did not include an overnight stay. Comorbidities were defined based on diagnosis codes in the primary or secondary care setting and by medications according to the National Prescription Register. The study index date constituted the time of the first recorded physician’s diagnosis of COPD during the enrollment timeframe.

### Statistical analysis

A sample size calculation conducted prior to the study indicated that 13,800 patients were required to detect a 4% difference between groups, with a power of 80% and an *α* level of 5% in the two-tailed test. The statistical analysis software used was “PC SAS for Windows” Version 9.4.

Patient demographics were described for COPD patients and the reference population stratified by gender (men vs. women). The *χ*^2^ test was used to compare the two (or more if the analysis was stratified) groups of patients, with the outcome (e.g., percentage of patients with COPD and comorbidities) as the dependent variable and the group variable gender as the independent variable. The test for finding a time trend in asthma was done by the Cochran–Armitage trend test, with the percentage of asthma as the dependent variable and year as the independent variable. The time to death from the index date was compared between genders using Cox regression, with time to death as the dependent variable, gender as the independent variable, and age as covariate. The patients were censored at the last known time alive or at the end of the study. The analysis was presented as HRs with 95% CI for the group and covariate. Exacerbations were analyzed by comparing the percentage of patients with an exacerbation using a *χ*^2^ test and by using a Cox regression, with the time to an exacerbation as the dependent variable and age as covariate. An analysis of covariance was used to compare the difference between the groups with respect to hospital visits and primary care visits, number of prescriptions, and time from asthma diagnosis to the index date, with age as covariate. Comorbidities were calculated at different time points as frequencies of the total study population at that time point. The overall comorbidity burden was assessed using the CCI.

### Reporting summary

Further information on research design is available in the Nature Research Reporting Summary linked to this article.

## Supplementary information

Supplementary Information

Reporting Summary

## Data Availability

The data for this study were obtained from EMRs in primary care and the Swedish National Health Register. Restrictions apply to the availability of these data, which were used under license for the current study, and so are not publicly available. Data are however available from IQVIA Solutions, Copenhagen, Denmark, upon reasonable request and with permission of the Swedish National Health Register.
