# Supplementary material for: Gender differences among Swedish COPD patients: results from the ARCTIC, a real-world retrospective cohort study
Source: NPJ Prim Care Respir Med. 2019 Dec 10;29:45. doi: 10.1038/s41533-019-0157-3 (PMC6904454; doi:10.1038/s41533-019-0157-3)
Supplement: Supplementary file 1 — Supplementary Information [file 41533_2019_157_MOESM1_ESM.pdf]

Supplementary Information

Gender differences among Swedish COPD patients: Results from the ARCTIC, a real-world retrospective cohort study

Karin Lisspers[1], Kjell Larsson[2], Christer Janson[3], Björn Ställberg[1], Ioanna Tsiligianni[4], Florian S. Gutzwiller[5], Karen Mezzi[5], Bine Kjoeller Bjerregaard[6], Leif Jorgensen[6], Gunnar Johansson[1]

[1]Department of Public Health and Caring Sciences, Family Medicine and Preventive Medicine, Uppsala University, Uppsala, Sweden; [2]Department of Pulmonary Medicine, Work Environment Toxicology, Karolinska Institutet, Stockholm, Sweden; [3]Department of Medical Sciences: Respiratory, Allergy and Sleep Research, Uppsala University, Uppsala, Sweden; [4]Department of Social Medicine, Health Planning Unit, Faculty of Medicine, University of Crete, Crete, Greece; [5]Novartis Pharma AG, Basel, Switzerland; [6]IQVIA Solutions, Copenhagen, Denmark

Corresponding author:

Dr. Karin Lisspers

Department of Public Health and Caring Sciences

Family Medicine and Preventive Medicine

Uppsala University, BMC, Box 564, SE-751 22

Uppsala, Sweden

Tel.: +46 241 498510; Fax: +46 184 716675

E-mail address: karin.lisspers@ltdalarna.se

Running head: Gender aspects of COPD in a Swedish cohort: the ARCTIC study

Contents

Supplementary Table 1. ICD-10 codes for diseases 3

Supplementary Table 2. ATC codes for medications 6

Supplementary Table 3. Difference between females and males in reason for mortality[a] 8

Supplementary Table 1. ICD-10 codes for diseases

Disease	ICD 10 codes
Asthma	J45/J46
Cardiovascular disease	I00-I99
Acute rheumatic fever	I00-I02
Chronic rheumatic heart diseases	I05-I09
Hypertensive diseases	I10-I15
Ischemic heart diseases	I20-I25
Pulmonary heart disease and diseases of pulmonary circulation	I26-I28
Other forms of heart disease	I30-I52
Cerebrovascular diseases	I60-I69
Diseases of arteries, arterioles and capillaries	I70-I79
Diseases of veins, lymphatic vessels and lymph nodes, not elsewhere classified	I80-I89
Other and unspecified disorders of the circulatory system	I95-I99
Diabetes Type I	E10
Diabetes Type II	E11, E13
Hyperlipidemia	E78.5
Depression	F32, F33
Major depressive disorder, single episode	F32
Major depressive disorder, recurrent	F33
Anxiety	F40, F41
Phobic anxiety disorders	F40
Other anxiety disorders	F41
Gastro-esophageal reflux disease (GERD)	K21
Gastro-esophageal reflux disease with esophagitis	K21.0
Gastro-esophageal reflux disease without esophagitis	K21.9
Chronic pain	G89.2, G89.4

Chronic pain, not elsewhere classified	G89.2
Chronic pain due to trauma	G89.21
Chronic post-thoracotomy pain	G89.22
Other chronic postprocedural pain	G89.28
Other chronic pain	G89.29
Chronic pain syndrome	G89.4
Dementia	F01, F02, F03
Vascular dementia	F01
Dementia in other diseases classified elsewhere	F02
Unspecified dementia	F03
Osteoporosis	M80, M81
Osteoporosis with current pathological fracture	M80
Osteoporosis without current pathological fracture	M81
Fractures	S2
Fracture of skull and facial bones	S02
Fracture of cervical vertebra and other parts of neck	S12
Fracture of rib(s), sternum and thoracic spine	S22
Fracture of lumbar spine and pelvis	S32
Fracture of shoulder and upper arm	S42
Fracture of forearm	S52
Fracture at wrist and hand level	S62
Fracture of femur	S72
Fracture of lower leg, including ankle	S82
Fracture of foot and toe, except ankle	S92
Lung cancer	C34
Any other cancer	C00-D49
Malignant neoplasms of lip, oral cavity and pharynx	C00-C14

Malignant neoplasms of digestive organs	C15-C26
Malignant neoplasms of respiratory and intrathoracic organs	C30-C39
Malignant neoplasms of bone and articular cartilage	C40-C41
Melanoma and other malignant neoplasms of skin	C43-C44
Malignant neoplasms of mesothelial and soft tissue	C45-C49
Malignant neoplasms of breast	C50
Malignant neoplasms of female genital organs	C51-C58
Malignant neoplasms of male genital organs	C60-C63
Malignant neoplasms of urinary tract	C64-C68
Malignant neoplasms of eye, brain and other parts of central nervous system	C69-C72
Malignant neoplasms of thyroid and other endocrine glands	C73-C75
Malignant neuroendocrine tumors	C7A
Secondary neuroendocrine tumors	C7B
Malignant neoplasms of ill-defined, other secondary and unspecified sites	C76-C80
Malignant neoplasms of lymphoid, hematopoietic and related tissue	C81-C96
In situ neoplasms	D00-D09
Benign neoplasms, except benign neuroendocrine tumors	D10-D36
Benign neuroendocrine tumors	D3A
Neoplasms of uncertain behavior, polycythemia vera and myelodysplastic syndromes	D37-D48
Neoplasms of unspecified behavior	D49
Rhinitis	J30, J31.0
Vasomotor rhinitis	J30.0
Chronic rhinitis NOS	J31.0
Allergic rhinitis due to pollen	J30.1
Other season allergic rhinitis	J30.2
Allergic rhinitis due to food	J30.5
Allergic rhinitis due to animal hair and dander (cat, dog)	J30.81

Other allergic rhinitis	J30.89
Allergic rhinitis, unspecified	J30.9
Nasal polyps	J33
Polyp of nasal cavity	J33.0
Polypoid sinus degeneration	J33.1
Other polyp of sinus	J33.8
Nasal polyp, unspecified	J33.9
Polymyalgia rheumatica	M35.3
Kidney disease	N17, N18, N19
Acute kidney failure	N17
Chronic kidney disease	N18
Unspecified kidney failure	N19
Rheumatoid arthritis	M05-M06
Rheumatoid arthritis with rheumatoid factor	M05
Other rheumatoid arthritis	M06
Respiratory diseases	J00-J99
Pneumonia	J12-J18
Respiratory tract infections	J00-J06
Influenza	J09-J11
Acute bronchitis	J20
Bronchiectasis	J47

ICD International Classification of Diseases

Supplementary Table 2. ATC codes for medications

Medication	ATC codes
Cardiovascular medication	C
Cardiac therapy	C01

Antihypertensives	C02
Diuretics	C03
Peripheral vasodilators	C04
Vasoprotectives	C05
Beta-blocking agents	C07
Combinations of beta-blocking agents and calcium channel blockers	C07F
Calcium channel blockers	C08
Agents acting on the renin-angiotensin system	C09
Combinations of agents acting on the renin-angiotensin system and diuretics	C09BA, C09DA
Combinations of agents acting on the renin-angiotensin system and calcium channel blockers	C09BB, C09DB
Lipid modifying agents	C10
Statins	C10AA, C10B
HMG CoA reductase inhibitors	C10AA
Lipid-modifying agents, combinations	C10B
Proton-pump Inhibitors	A02
Antidepressants	N06A
Anxiety	N05B
Central pain killers	
Propionic and acid derivatives	M01AE
Opioids	N02A
Anilides	N02BE
Sleep medication	N05C
Bisphosphonates	M05BA
Nasal corticosteroids	R01AD
Diabetes medication	A10

SABA	R03AC02, R03AC03, R03CC02, R03CC03
LABA	R03AC12, R03AC13, R03AC18, R03AC19 R03CC12
LAMA	R03BB04, R03BB05, R03BB06, R03BB07
Combination LABA + LAMA	R03AL03, R03AL04, R03AL05, R03AL06
Combination ICS + LABA	R03AK (R03AK06, R03AK07, R03AK08, R03AK10, R03AK11)
ICS	R03BA
Oral steroids	H02AB

ATC Anatomical Therapeutic Chemical, *HMG CoA* 3-hydroxy-3-methylglutaryl coenzyme A, *ICS* inhaled corticosteroids, *LABA* long-acting β_2-agonist, *LAMA* long-acting muscarinic antagonist, *SABA* short-acting β_2-agonist

Supplementary Table 3. Difference between females and males in reason for mortality[a]

Disease	Male (*N* = 8,593)	Female (*N* = 9,993)
Reason for the death, on high group level		
I - Diseases of the circulatory system	776 (9.03)	671 (6.71)
C - Neoplasms	552 (6.42)	528 (5.28)
J - Diseases of the respiratory system	445 (5.18)	473 (4.73)
K - Diseases of the digestive system	76 (0.88)	78 (0.78)
R - Symptoms, signs and abnormal clinical and laboratory findings, not elsewhere classified	50 (0.58)	52 (0.52)
X - External causes of morbidity and mortality	60 (0.70)	29 (0.29)
E - Endocrine, nutritional and metabolic diseases	46 (0.54)	38 (0.38)
F - Mental and behavioral disorders	29 (0.34)	48 (0.48)
A - Certain infectious and parasitic diseases	27 (0.31)	42 (0.42)
G - Diseases of the nervous system	28 (0.33)	24 (0.24)
N - Diseases of the genitourinary system	25 (0.29)	25 (0.25)

D - Neoplasms	19 (0.22)	21 (0.21)
W - External causes of morbidity and mortality	19 (0.22)	17 (0.17)
B - Certain infectious and parasitic diseases	15 (0.17)	15 (0.15)
Y - External causes of morbidity and mortality	11 (0.13)	10 (0.10)
M - Diseases of the musculoskeletal system and connective tissue	9 (0.10)	10 (0.10)
D - Diseases of the blood and blood-forming organs and certain disorders involving the immune mechanism	9 (0.10)	6 (0.06)
L - Diseases of the skin and subcutaneous tissue	4 (0.05)	2 (0.02)
Q - Congenital malformations, deformations and chromosomal abnormalities	1 (0.01)	2 (0.02)
V - External causes of morbidity and mortality	2 (0.02)	1 (0.01)
Reason for the death, on 3-character level		
J44 - Other chronic obstructive pulmonary disease	345 (4.01)	387 (3.87)
C34 - Malignant neoplasm of bronchus and lung	198 (2.30)	212 (2.12)
I25 - Chronic ischemic heart disease	196 (2.28)	120 (1.20)
I21 - ST elevation (STEMI) and non-ST elevation (NSTEMI) myocardial infarction	155 (1.80)	121 (1.21)
I50 - Heart failure	68 (0.79)	82 (0.82)
I48 - Atrial fibrillation and flutter	53 (0.62)	48 (0.48)
C61 - Malignant neoplasm of prostate	79 (0.92)	-
I71 - Aortic aneurysm and dissection	44 (0.51)	32 (0.32)
C25 - Malignant neoplasm of pancreas	35 (0.41)	37 (0.37)
R99 - Ill-defined and unknown cause of mortality	35 (0.41)	32 (0.32)
C18 - Malignant neoplasm of colon	27 (0.31)	39 (0.39)
F03 - Unspecified dementia	18 (0.21)	42 (0.42)
J43 - Emphysema	30 (0.35)	30 (0.30)
I63 - Cerebral infarction	32 (0.37)	25 (0.25)
E14 - E14	29 (0.34)	24 (0.24)

A41 - Other sepsis	17 (0.20)	32 (0.32)
I64 - I64	21 (0.24)	27 (0.27)
I70 - Atherosclerosis	25 (0.29)	22 (0.22)
J18 - Pneumonia, unspecified organism	29 (0.34)	18 (0.18)
C80 - Malignant neoplasm without specification of site	17 (0.20)	28 (0.28)
I69 - Sequelae of cerebrovascular disease	14 (0.16)	29 (0.29)
C50 - Malignant neoplasm of breast	-	42 (0.42)
X59 - X59	29 (0.34)	12 (0.12)
C22 - Malignant neoplasm of liver and intrahepatic bile ducts	24 (0.28)	15 (0.15)
I51 - Complications and ill-defined descriptions of heart disease	18 (0.21)	20 (0.20)
I35 - Nonrheumatic aortic valve disorders	23 (0.27)	13 (0.13)
C67 - Malignant neoplasm of bladder	23 (0.27)	12 (0.12)
I61 - Nontraumatic intracerebral hemorrhage	20 (0.23)	13 (0.13)
I11 - Hypertensive heart disease	13 (0.15)	15 (0.15)
K70 - Alcoholic liver disease	15 (0.17)	12 (0.12)
C15 - Malignant neoplasm of esophagus	17 (0.20)	9 (0.09)
R54 - Age-related physical debility	7 (0.08)	19 (0.19)
W19 - Unspecified fall	11 (0.13)	15 (0.15)
C16 - Malignant neoplasm of stomach	8 (0.09)	17 (0.17)
C20 - Malignant neoplasm of rectum	18 (0.21)	6 (0.06)
I26 - Pulmonary embolism	13 (0.15)	11 (0.11)
J84 - Other interstitial pulmonary diseases	15 (0.17)	6 (0.06)
K55 - Vascular disorders of intestine	7 (0.08)	13 (0.13)
C64 - Malignant neoplasm of kidney, except renal pelvis	11 (0.13)	8 (0.08)
I20 - Angina pectoris	12 (0.14)	7 (0.07)
G30 - Alzheimer's disease	8 (0.09)	10 (0.10)

I42 - Cardiomyopathy	9 (0.10)	8 (0.08)
I67 - Other cerebrovascular diseases	10 (0.12)	7 (0.07)
I27 - Other pulmonary heart diseases	6 (0.07)	10 (0.10)
K92 - Other diseases of digestive system	8 (0.09)	8 (0.08)
C56 - Malignant neoplasm of ovary	-	15 (0.15)
C85 - Other specified and unspecified types of non-Hodgkin lymphoma	8 (0.09)	7 (0.07)
I10 - Essential (primary) hypertension	6 (0.07)	9 (0.09)
J45 - Asthma	3 (0.03)	12 (0.12)
K56 - Paralytic ileus and intestinal obstruction without hernia	8 (0.09)	7 (0.07)
C92 - Myeloid leukemia	9 (0.10)	5 (0.05)
D38 - Neoplasm of uncertain behavior of middle ear and respiratory and intrathoracic organs	7 (0.08)	7 (0.07)
B99 - Other and unspecified infectious diseases	6 (0.07)	7 (0.07)
C71 - Malignant neoplasm of brain	5 (0.06)	8 (0.08)
C90 - Multiple myeloma and malignant plasma cell neoplasms	7 (0.08)	6 (0.06)
J98 - Other respiratory disorders	3 (0.03)	10 (0.10)
E11 - Type 2 diabetes mellitus	7 (0.08)	5 (0.05)
I13 - Hypertensive heart and chronic kidney disease	7 (0.08)	5 (0.05)
C43 - Malignant melanoma of skin	8 (0.09)	3 (0.03)
C91 - Lymphoid leukemia	9 (0.10)	2 (0.02)
D37 - Neoplasm of uncertain behavior of oral cavity and digestive organs	3 (0.03)	8 (0.08)
X70 - X70	8 (0.09)	3 (0.03)
G12 - Spinal muscular atrophy and related syndromes	3 (0.03)	7 (0.07)
I34 - Nonrheumatic mitral valve disorders	2 (0.02)	8 (0.08)
J69 - Pneumonitis due to solids and liquids	7 (0.08)	3 (0.03)

N19 - Unspecified kidney failure	6 (0.07)	4 (0.04)
N39 - Other disorders of urinary system	7 (0.08)	3 (0.03)
C26 - Malignant neoplasm of other and ill-defined digestive organs	4 (0.05)	5 (0.05)
C76 - Malignant neoplasm of other and ill-defined sites	5 (0.06)	4 (0.04)
I08 - Multiple valve diseases	6 (0.07)	3 (0.03)
I73 - Other peripheral vascular diseases	4 (0.05)	5 (0.05)
K74 - Fibrosis and cirrhosis of liver	4 (0.05)	5 (0.05)
N18 - Chronic kidney disease (CKD)	4 (0.05)	5 (0.05)
C24 - Malignant neoplasm of other and unspecified parts of biliary tract	1 (0.01)	7 (0.07)
F10 - Alcohol-related disorders	5 (0.06)	3 (0.03)
K85 - Acute pancreatitis	4 (0.05)	4 (0.04)
C49 - Malignant neoplasm of other connective and soft tissue	4 (0.05)	3 (0.03)
C83 - Non-follicular lymphoma	5 (0.06)	2 (0.02)

[a]Percentage calculated relative to the population at baseline
